# What Do Community Doulas Think About the Future of the Doula Workforce?

**DOI:** 10.1007/s10995-024-03943-1

**Published:** 2024-07-24

**Authors:** Nika Darvish, Anu Manchikanti Gómez, Cassondra Marshall, Raichal McDonald, LaToshia Rouse, Lauren Dinsmore, Hannah Hecht, Ruth Berhanu, Grace Rajan, Jaspal Sandhu

**Affiliations:** 1 School of Public Health, University of California, Berkeley, Berkeley, CA USA; 2 Sexual Health and Reproductive Equity Program, School of Social Welfare, University of California, Berkeley, Berkeley, CA USA; 3 Giving Austin Labor Support, Austin, TX USA; 4 Birth Sisters Doula Services, Raleigh, NC USA; 5 School of Public Health, Boston University, Boston, MA USA

**Keywords:** Doulas, Health equity, Childbirth, Health futures, Technology

## Abstract

**Introduction:**

Expanding access to doula care is a key strategy for improving the perinatal experiences and health outcomes of birthing people of color in the U.S. This study investigates the future of maternal healthcare in the U.S. from the perspective of doulas and highlights emerging technology and other opportunities related to strengthening the doula workforce.

**Methods:**

The study recruited community doulas from 12 unique U.S. states, ensuring at least half of the doulas predominantly served communities of color. Doulas (N = 26) participated in semi-structured, futures-oriented interviews that explored their experiences providing care during the COVID-19 pandemic and utilization of technology. A subset of doulas (n = 8) were engaged in interactive workshops where they envisioned alternative futures for doula care and childbirth. Interviews and workshops were analyzed using the Framework Method.

**Results:**

The COVID-19 pandemic heightened technology use among doulas and increased client accessibility. Social media serves as a unique space for critical community building and client outreach. Doulas reported opportunities to strengthen and mobilize the future workforce: recognizing doula care as a reimbursable service by health insurers, utilizing doula collectives for community practice to decrease burnout, increasing emotional support for doulas, and instilling a chain of learning through mentorship.

**Discussion:**

Futures thinking served as a valuable approach for doulas to illuminate the implications of present-day challenges and empowered doulas to design roadmaps toward better futures for doulas and maternal health. Doulas should be engaged as partners to hold a meaningful decision-making role when discussing policies, employment structures, emerging technology, and other aspects of doulas’ positioning within the healthcare system.

## Significance

Doulas provide information, and emotional and physical support to birthing persons throughout labor, birth, and postpartum. Various studies have shown that doulas help to address gaps in maternal healthcare and reduce racial health disparities. Despite doulas’ impactful role in facilitating empowered childbirth experiences, they are often left out of decision-making conversations among maternal health professionals. By gathering doula perspectives on the future of maternal healthcare through blending futures thinking and racial equity, this study provides opportunities around emerging technology and other strategies to mobilize the doula workforce.

## Introduction

Racial disparities in birth outcomes between birthing people^1^ of color and white birthing people in the United States are well documented. Black and American Indian/Alaska Native (AIAN) birthing people are three to four times more likely than non-Hispanic white people to die from pregnancy-related causes (Howell, 2018), regardless of socioeconomic status (Creanga et al., 2017). Additionally, infants born to Black and AIAN birthing people face significantly higher mortality rates compared to those born to white birthing people (Pham et al., 2020). These disparities in maternal and infant health outcomes may be attributed to historical reproductive oppression, which has embedded racism into societal structures (Taylor, 2020). The persistence of structural racism’s impact is evident as maternal deaths continued to rise during the COVID-19 pandemic, more significantly among Black and Hispanic birthing people, compared to their non-Hispanic white counterparts (U.S. GAO, 2022).

Doulas, trained paraprofessionals who provide non-clinical support to birthing people throughout the perinatal period, are one strategy to reduce racial disparities in maternal health outcomes, primarily through a reduction in cesarean births, decreased use of epidurals, a reduction in preterm birth rates, and an increase in overall birth satisfaction (Bohren et al., 2017; Kozhimannil et al., 2013). Additionally, the *Safe Birth Checklist*, developed by the World Health Organization (WHO), recognizes doula support as an essential maternal and perinatal care practice (WHO Safe Childbirth Checklist, 2015). Different types of doulas offer specialized assistance. For example, full-spectrum doulas provide support across all reproductive experiences and life transitions, unlike traditional doulas who focus solely on childbirth. Additionally, birth and bereavement doulas specialize in supporting those who have experienced pregnancy or infant loss.

Community doulas further enhance this support by serving as culturally congruent health workers, often sharing similar lived experiences and cultural, racial, and other intersectional identities with their clients. In the prenatal and postpartum periods, community doulas assist with the navigation of health care and social services, provide peer education, and give social support. During labor, they provide a continuous-presence to promote physical comfort and support the birthing person’s emotional needs. Prior research has highlighted how community doula support leads to enhanced knowledge about childbearing, positive impacts on supportive networks, higher rates of breastfeeding initiation, and more positive parent-infant interactions amongst underserved birthing persons (Breedlove, 2005; Edwards et al., 2013; Hans et al., 2013).

The use of doulas, however, is not yet widespread. While data on the current utilization of doulas are limited, one retrospective analysis of birthing people nationally who underwent childbirth in 2011–2022 (N = 2400) found that 6% reported doula care during childbirth (Kozhimannil et al., 2014). The majority of doulas do not come from low-income communities or communities of color. Demographic data for doulas in the United States are limited. A survey conducted as part of the National Health Law Program’s advocacy for Medicaid coverage for doula care in California found that while doulas of color make up a substantial portion of the respondent pool, they are underrepresented relative to their clients and the overall birthing population (Chen & Robles-Fradet, 2020). This highlights the critical need for more doulas of color to better align with and serve the diverse racial and ethnic backgrounds of birthing people.

The outbreak of the COVID-19 pandemic catalyzed a surge in healthcare technology investments (Bestsennyy et al., 2022). From 2014 to 2018, more than 580 healthcare technology deals were made in the U.S., valued at $83 billion (Singhal et al., 2022). Despite women historically utilizing healthcare services, particularly preventative care services, more than their male counterparts, investments in digital health focusing on the U.S. female population only made up 7% of all digital health innovation funding in 2021 according to a report by Rock Health (DeSilva & Krasniansky, 2021; Vaidya et al., 2012).

The disproportionately low investment in digital health for the female population not only neglects doula support, still considered 'fringe' in many places in the United States, but also exacerbates existing issues in health outcomes and access. This underrepresentation underscores the untapped potential for technology to revolutionize doula care. To date, there are five mobile applications with five or more ratings available for both iPhone and Android users that specifically target doula work. Three out of five of the applications were developed within the last two years. Specific uses for these apps include client management for birth workers (Mobile Doula), connecting birthing people to birth workers and their services (Meela), and virtual, on-demand perinatal informational support from a doula’s perspective (Poppy Seed Health, eDoula, Naked Doula App). Other recent initiatives leveraging technology to mobilize doula care include startup Brave Health’s partnership with The Doula Network (TDN) to improve the maternal health experience of birthing people on Medicaid by aligning doulas, clients, health plans, and technology (Landi, 2022).

A key barrier to access is the cost of hiring a doula. Most clients pay doulas out of pocket, with services ranging from several hundred dollars to over $2000 (Chen & Robles-Fradet, 2020). Insurance reimbursement for doula care is rare among private insurance. States are increasingly seeking federal authorization to provide doula services as an optional benefit under their state Medicaid programs to pregnant beneficiaries. As of April 2024, 13 U.S. states use a variety of approaches to provide doula services within their Medicaid programs, up from just 3 before 2021 (Hasan, 2022). Despite policy advancements, the high cost of doulas and relatively limited coverage by state Medicaid programs exclude low-income birthing people from accessing doula support.

## Objectives/Purpose

This study investigates the future of maternal healthcare in the U.S. from the doula’s perspective by engaging doulas in future-oriented interviews and workshops. Futures studies is a framework used to postulate possible, probable, and preferable futures. Studies designed to understand health futures often require futures thinking, a mindset that involves envisioning desirable futures and creating roadmaps for practice by aligning key stakeholders (Buehring & Liedtka, 2018; Institute for the Future, 2022). A variety of multidisciplinary methods can be used to practice futures thinking. Visioning is a process of imagining and articulating possible future scenarios or outcomes. Backcasting starts from a desired future or outcome and works backward to identify the steps needed to reach that goal (Garrett, 1999).

Given a doula’s intimate role in multiple stages of a birthing person’s perinatal journey, stories from the doula’s perspective provide insight into opportunities for improving maternal healthcare practice and policies, as well as a better understanding of a doula’s role and impact during childbirth. The objective of this study is to explore emerging opportunities to mobilize the doula workforce in the U.S. The intended audience of this work includes policymakers, doulas, technologists, healthcare providers, payers, and decision-makers within large healthcare systems.

## Methods/Description

A demographic questionnaire and semi-structured interview guide were developed based on the current literature (Wint et al., 2019). Futures-oriented interviews (N = 26) were designed to capture maximum information about doulas’ experiences working with clients, the challenges of providing services during the COVID-19 pandemic, their use of technology, and their perceptions of doula care and childbirth futures.

A combination of purposive and snowball sampling was used to recruit doulas for participation. Doulas were recruited through an online survey posted to doula nonprofits, doula certification databases, and social media platforms—primarily Facebook Groups. We followed up with survey respondents via email to participate in one-on-one interviews. Interviews lasted forty-five minutes on average. Doulas provided informed consent before their inclusion in the study.

After one-on-one interviews were conducted, a subset of doulas (n = 8) engaged in future-oriented workshops selected via a two-step sampling process. First, we filtered doulas into two groups based on the depth of responses to determine the high potential for contribution to the workshops. We then completed maximum variation sampling based on geography (Palinkas et al., 2015). Futures workshops consisted of ninety-minute interactive sessions where doulas envisioned alternate futures for doula work and childbirth by the year 2040. Three workshops were held in groups of two or three in sequential order and facilitated by research personnel. Each workshop occurred once. Interviews and workshops were conducted virtually and were audio-recorded, transcribed verbatim, and analyzed using the Framework Method, a systematic approach to qualitative data analysis that involves organizing and analyzing data according to predefined themes or categories (Gale et al., 2013). Semi-structured interviews were conducted until thematic saturation was achieved.

We primarily recruited community doulas who provide labor, postpartum, full-spectrum, and birth and bereavement support. We ensured that at least half of doulas offered a significant portion of services at a low or no cost. We also ensured that at least half of doulas primarily provided services to communities of color.

Each transcript was coded by a single reviewer using a codebook developed and iterated through a two-stage process (Ando et al., 2014). The first stage consisted of an inductive approach to develop a codebook from six transcripts. Researchers identified patterns, themes, and recurring concepts within the transcripts based on the content to develop a list of initial codes. Once a substantial number of codes were generated, they were grouped into broader, high-level themes. The codebook was then applied to the remaining transcripts for iterations of sub-codes, where code names were modified as needed and new codes were added as nuanced concepts emerged. The two-tier codebook consisted of ten high-level themes, each with 2–17 sub-codes. The codebook served as a foundational tool within the Framework Method, guiding the systematic coding and organization of interview data and facilitating the analysis and interpretation of key themes and patterns.

Additionally, we included an advisory committee made up of birth and postpartum doulas (R.M., L.R.) and doula researchers with a focus on health equity (J.S., C.M., A.G.). Doula advisors were interviewed and selected from among applicants (N = 41) from across the United States. Advisory committee members held a leadership role in shaping the project’s direction and contextualizing study findings. Based on their input, the interview guide was modified to include 'what if' and 'I wish' prompts. Workshops were restructured to integrate doulas’ insights on envisioning healthy futures for childbirth, in addition to doula work. The study duration lasted from March 2021 to May 2022. The research was approved by the Institutional Review Board at the University of California, Berkeley.

## Results

All doulas interviewed (N = 26; *M*_age_ = 34) had experience working with at least two clients. Fifty percent (50%) of doulas self-identified as non-Hispanic Black or African American, 27% as non-Hispanic white, 11% as Hispanic, 8% as Asian, and 4% as non-specific. Roughly half of the doulas reported 70% or more of their clientele as low-income. Eighty-five percent (85%) of doulas interviewed reported being a community doula, and more than half of doulas reported practicing for five or more years. In this paper, we will refer to the sample and findings as “community doulas.” The demographic characteristics of the sample are included in (Table 1).

**Table 1:** Participating doula demographics (N = 26)

Characteristic		N	(%)
Race/Ethnicity	Non-Hispanic Black or African American	13	50
	Non-Hispanic white	7	27
	Hispanic	3	11
	Asian	2	8
	Non-specific	1	4
Community doula	Yes	22	85
	No	4	15
Years of experience	≥ 5 years	14	54
	< 5 years	12	46
Doula primary type (N = 21)	Birth	9	43
	Birth and Postpartum	6	29
	Full Spectrum	3	14
	Postpartum	2	10
	Birth and Bereavement	1	4
Low-income clientele percentage (N = 25)	≥ 70%	11	44
	30%–70%	8	32
	≤ 30%	6	24
Age	28–40	11	42
	≤ 28	8	31
	≥ 40	7	27

Several major findings related to our aims emerged after the analysis of one-on-one interviews that were further supported by outcomes from futures workshops. We present the major findings below under three main themes: (1) Technology, Both a Facilitator and a Barrier; (2) Building Holistic Healthcare Ecosystems: The Impact of Empowered Doula-Community Partnerships; and (3) Sustainable Doula Futures: Nurturing Resilient Doulas Through Inclusive Coverage and Community. The major themes and subthemes are listed in (Table 2).

**Table 2:** Themes and subthemes

Theme	Subthemes
Technology, Both a Facilitator and a Barrier	Social Media Serves Multiple Critical Functions
	Virtual Support Increases Client Accessibility and Equity
	Limitations of Technology-Facilitated Doula Care During the COVID-19 Pandemic
Building Holistic Healthcare Ecosystems: The Impact of Empowered Doula-Community Partnerships	Empowering Doulas Empowers Communities
	Doulas Endorse Intersectional Collaboration with Clinicians
Sustainable Doula Futures: Nurturing Resilient Doulas Through Inclusive Coverage and Community	Covering Doula Care Through Private Health Insurance
	Stronger Together: Doula Community Building

### Technology, Both a Facilitator and a Barrier

Doulas’ perspectives on technology clustered into three main subthemes: ’Social Media Serves Multiple Critical Functions,” “Virtual Support Increases Client Accessibility and Equity,” and “Limitations of Technology-Facilitated Doula Care During the COVID-19 Pandemic.” Comments from doulas are referenced using anonymous identifiers.

#### Social Media Serves Multiple Critical Functions

Doulas primarily use social media for client outreach and community building. Instagram and Facebook are the top two social media platforms doulas use for client outreach, promotional marketing, and advertising **(**Table 3**).** Technology platforms not related to social media that were discussed by doulas in interviews are listed in (Table 4). One doula mentioned how they, “wouldn’t be able to do my work without technology, without social media and all of that, without my Instagram to post things about what I’m doing, it’s invaluable.”—Doula O, [California].

**Table 3:** High-frequency technology modalities mentioned during interviews

Tech modality	Use associated with tech modality mentioned	Count (n)	Frequency (%)
Cell Phone	General communication with clients	17	65
	Client billing	2	8
	Playing music during birth	1	4
	Taking photos during birth	1	4
Facebook—Groups	Informational, emotional, and general support groups for birth workers	13	50
Zoom	Client consultations	12	46
	Doula training webinars	4	15
	Informational birth classes	2	8
Instagram	Client outreach, promotional marketing, advertising	10	38
	Networking with other birth workers	4	15
	Instagram Live	1	4
	Source for clinical information	1	4
Email	General communication with clients	8	31
	Communication with other birth workers	1	4
Personal Website	Client outreach, promotional marketing, advertising	7	27
Facebook—General Platform	Client outreach promotional marketing, advertising	5	19
Facetime	Client Consultations	5	19
Contraction Timing App	Timing contractions during labor	3	12
Google Docs	Preparing teaching materials and client tracking	3	12

**Table 4:** Non-social media technology platforms and purpose(s) mentioned ≥ 2 times during interviews

Technology platform mentioned	Purpose(s) of utilization
Dropbox, Honeybook, Mobile Doula	Client charting and tracking
Same Page, GroupMe, LinkedIn	Communicating and networking with other birth workers
Craigslist.com, Doulamatch.net	Client outreach, promotional marketing, and advertising
Acuity, Calendly	Scheduling client consultations
Canva	Creating outreach materials digitally
Headspace	Breathing exercises and body relaxation techniques
Humana	Client Billing

Virtual Facebook Groups were specifically mentioned in thirteen out of twenty-six interviews as being a vital space to exchange information and advice amongst other birth workers. Doulas described how the virtual communities offer a place to seek guidance and receive support from their peers:

“I’m in a bunch of doula groups on Facebook right now. I definitely use those to gain more information. I recently asked a question in one of them about a client just to confirm the information I was going to give her was correct. Sure enough, the fellow doulas did confirm what I was going to say, but it was great having that space and being able to bounce some things off fellow birth workers.”—Doula N, [Washington]

In addition to an informational forum, doulas shared how social media platforms provide a unique space for community building and social support. One doula mentioned,

“Networking has been great, whether it’s through LinkedIn, or other social media, it’s been pretty incredible. I feel like I have all these doula friends just from social media. I’ve never met any of these women in person, but it’s nice to have those communities on our little phones and computers.”—Doula T, [Pennsylvania]

Another doula reflects on the role of social media in helping doulas stay informed, confident in their practice, and connected to the broader network of birth workers, ultimately enhancing the quality of care they provide to their clients by fostering collaboration,

“Online spaces are where we depend on one another to kind of bounce ideas off each other. It’s like, ‘Do you have any experience with a mom where this or that is going on?’ Or, ‘Something is happening in my life and I’m on call for a birth right now, can you back me up?’ The online communities have been extremely beneficial.”—Doula A, [Alabama]

#### Virtual Support Increases Client Accessibility and Equity

Another theme that emerged from the doulas’ discussion on technology was client accessibility. When discussing how they leveraged technology to provide community doula care during shelter-in-place restrictions during the COVID-19 pandemic, doulas reported increased use of Zoom and Facetime, which enabled them to cast a wider net of clients. Doulas described the advancement as follows:

“It [technology] has greatly impacted my work now because I see a lot of clients virtually. Especially when the pandemic first happened, a lot of doulas were scared to go to homes, and a lot of us weren’t even allowed in hospitals. It was a lot of testing and virtual meetings. Now, all my consultations are online. It’s a lot easier, that way we don’t have to be in contact in person to see if we are vibing or not. That also allowed me to reach a lot more clients than I was before having to go out of the home and to the hospital and doctor’s appointments.”—Doula H, [California]

“It [technology] has been everything. I take clients all over the place. I sometimes would drive for two hours to get to a session that only lasts an hour and then spend another two hours going home, so I eat my whole day just to do a one-hour visit. It has been nice to do all of this, especially the weekly support, virtually.” —Doula D, [California]

Another doula highlighted how technology enables the maintenance of doula care despite restrictions set in place by healthcare centers:

“I mean, with COVID it [technology] has allowed us to have access to spaces when we were kicked out of the hospitals. I mean, texting, emailing, FaceTime, just having more options for the ability to provide support and care."—Doula V, [Texas]

When asked how technology can be leveraged by the year 2040 during futures workshops, doulas’ responses supported the use of technology as a tool for making community doula care, such as client consultations, birth planning sessions, and birth classes, more accessible. Another doula who participated in the workshop expanded on their wish for technology to support equity:

“When it comes to technology, right now I’m participating in childbirth education and it’s with people of every walk of life, and it’s virtual. People can tap in as needed; we’ve done everything virtually except comfort measures, and some births I’ve attended virtually. I think technology will make things more accessible, therefore, hoping to make it more equitable.”—Doula A, [Alabama]

#### Limitations of Technology-Facilitated Doula Care During the COVID-19 Pandemic

While doulas agree that technology leverages accessibility to community doula care, they also revealed limitations to its use for in-person services. As the following quotes reflect, the major aspect of this theme includes the irreplaceability of human touch, physical interactions, and intuition. During interviews, doulas spoke about the challenges of providing services virtually:

“But without it [technology], I wouldn’t have been able to be present at all. And so that’s the part of technology I’m grateful for. But you can’t put emotion through a video. I can try to convey it as much as I possibly can, but a doula’s presence is what brings about the experience that a woman or a birth person needs at that moment, just that reassurance… you can’t do that through video. You have to physically be there.”—Doula F, [Minnesota]

“It [technology] has also taken away some personal or interpersonal communication. So once COVID happened and I started meeting clients virtually or through telephone, just like being in someone’s presence, something was missing. I’m not able to touch my clients. When I’m teaching comfort measures, I’d rather be there so that they can feel it. For example, teaching a partner how to use these comfort measures on a pregnant individual is a little difficult to show through a Zoom call or explain over the phone.”—Doula R, [New Jersey]

Another stated during a futures workshop:

“So much of what I do, I do intuitively based on what I’m seeing from a birthing person as they’re moving about a space. How are they breathing? Are they walking? How are they lifting their heel? What is their body motion telling me about what needs to happen with their baby? And while I can make that work and have made it work over the last two years, having to be virtual, it’s not ideal. It’s not the best doula support that I can offer, but it’s better than nothing. And so unfortunately, there will always be a population of people for whom better than nothing is good, but wouldn’t it be nice if we didn’t have to do that.”—Doula D, [California]

### Building Holistic Healthcare Ecosystems: The Impact of Empowered Doula-Community Partnerships

In exploring the future landscape of community doula care, the narratives shared by community doulas resonated with the role of doulas evolving beyond individualized support during childbirth to encompass broader community empowerment. As we explore these narratives, it becomes evident that the presence of doulas not only enhances the birthing journey but also lays the foundation for holistic healthcare ecosystems. This section delves into the influence of empowered doula-community partnerships on maternal well-being, societal health, and collaborative healthcare practices.

#### Empowering Doulas Empowers Communities

Continuous support from doulas during childbirth leads to empowered birthing experiences, which subsequently empower future generations. During interviews, doulas describe that if every birthing person was provided a doula:

“I think we’d have more empowered experiences, more educated experiences. I think it would have a huge impact on maternal mortality, a huge impact on postpartum depression, a huge impact on just moms not feeling like they’re just getting by, but they’re enjoying their postpartum. They’re enjoying their pregnancy. It’s not so cut and dry, like, okay, you go to the hospital, you have your baby, maybe you go home and nobody talks to you again for six weeks. Good luck. Maybe this improves postpartum care in general.”—Doula U, [Hawaii]

The positive, long-term effects of an empowered birth experience are highlighted by several doulas:

“I feel like women will be more empowered as a whole. I feel like especially women that need it the most, which are pregnant women, that they’re going through a lot. I feel like as a population we would be healthier.”—Doula Q, [North Carolina]

“Birth is going to change the way she interacts with her child. That’s going to change that child’s relationship with their mother and themselves. That’s going to create a stronger human being. And if we had a world of those, we would have less anxiety, less stress, more joy, and more laughter. That’s what a powerful birth can do, is create strong connections between the mother and the child so that the child grows up with that sense of strong connection, safety, and security. That can absolutely have lifelong effects.”—Doula C, [California]

#### Doulas Endorse Intersectional Collaboration with Clinicians

Intersectional collaboration between doulas and clinicians was another subtheme in doulas’ idealized futures. The following quotes illuminate doulas’ visions for integration within the clinical healthcare ecosystem. One doula reflects on the complementary care doulas provide alongside clinicians:

“I wish that more doctors and nurses would be exposed to the information and the education that doulas get. Even though their job is not to provide the same kind of support and care that a doula does, they at least know what it is that we do exactly. They are aware and have some of the same knowledge that we have as far as manipulating the baby and not having it be so much like a surgical procedure. To see women who are pregnant, not as someone who’s coming to the hospital with an illness, but as something that naturally happens for birthing persons.”—Doula R, [New Jersey]

Another suggests making doula care a customary practice among healthcare centers:

“Perhaps it would look like hospitals having doulas on call. OB-GYN offices could have a bank of doulas to select from. Folks could opt into that or opt out and they’d have that support. It would just be more accessible.”—Doula J, [California]

Lastly, one doula offers opportunities for storytelling to bridge disconnects between clinicians and doulas:

“It would be helpful if we brought the groups [clinicians and doulas] together more often to share stories to understand each other better. Hopefully, in doing that, they understand us better as well and start recognizing us as a team member and advocate, not an adversary. And I think that ultimately makes for more positive birth experiences, when the people on the team are all working together agreeably.”—Doula F, [Minnesota]

### Sustainable Doula Futures: Nurturing Resilient Doulas Through Inclusive Coverage and Community

#### Covering Doula Care Through Private Health Insurance

In envisioning a more inclusive and supportive healthcare landscape, doulas describe a pivotal shift in the perception and policy regarding doula care. Integrating doula services into health insurance plans marks a shift from the traditional model, advocating for broader accessibility and recognition of the invaluable role doulas play in maternal health:

“Doulas should be a part of our health insurance plan. It’s not going to be a la carte anymore. It’s not going to be self-paid anymore. It is going to be automatic, just like they brought that gym membership in with your insurance as an incentive, a doula will be an incentive as well.”—Doula Y, [Florida]

Others underscore the evolving landscape of reimbursement for doula care, pointing towards a potential shift where private insurance companies may follow suit:

“Medicaid’s a huge focus right now just to be able to offer more BIPOC clients doula services, where doulas aren’t having to take on clients pro bono or low cost. I expect that more private insurance companies might start reimbursing doulas, which is a good thing, but it may also introduce this whole new area for doulas having to contract with insurance companies and request reimbursement. I feel like that’s going to happen at some point in the future.”—Doula P, [Washington]

However, as she suggests, the transition may introduce new administrative complexities for doulas, signaling both opportunities and challenges on the horizon.

#### Stronger Together: Doula Community Building

One doula shared their vision for collaborative care within the doula community, highlighting a recent experience with a co-doula arrangement. This innovative approach not only enhanced support for the birthing family but also fostered sustainability and camaraderie among doulas. Her reflections highlight the potential for such community-based models:

“I would love to see more community building with my peers. I did a birth recently where I was a co-doula. It was two of us who worked with the same family the whole time, rather than a backup situation. We did all our prenatal and postpartum visits together. With the birth, we were able to switch back and forth. At 14 hours, we clock out, next person comes in, clock out, next person comes in. It was really helpful and felt very sustainable. I may move more into a community kind of model in terms of providing care different from community-based care, but in a similar vein like that."—Doula V, [Texas]

While others shared their aspiration for the future of doula care, they emphasized the importance of mentorship and education within their respective communities, highlighting their critical role in nurturing the next generation of doulas and advancing holistic maternal healthcare:

*“*I hope to be able to have the space to pass down the knowledge that I was granted by my teachers and my community to keep that community and communal learning lineage going. I envision myself as a mentor to a group of doulas, or even mental health workers. I really am in a place of pushing for more awareness for perinatal mental health needs because like I mentioned, the disparities are great.”—Doula K, [California]

“In the future, I see myself focusing more on the childbirth and education aspect of it than being exactly in the hospital, because my goal is for us to train more doulas, making an avenue and pathway for women to take this on as a career.”—Doula G, [Mississippi]

In addition to community practice and training doulas through a mentorship model, doulas also recognized the lack of emotional support for doulas and its vital role in order to keep the profession sustainable. One doula emphasized the need for recognition and support within the doula community, emphasizing the importance of acknowledging the toll that the work takes on individuals and their families:

“I hope that they realize that we are people. Although we chose to do this work, it’s very taxing. It’s really hard on our partners. It’s nice to be compensated for what we do and be respected and cared for as well by our community. I think that’s really important to do in the future.”—Doula H, [California]

In envisioning a future where doulas are valued and cared for by their communities, others advocated for a holistic approach that prioritizes the well-being of doulas:

“Many doulas experience secondary trauma or vicarious trauma either from their client stories or from being there in person and having no one to talk to about it. I think building those support networks is really important for the future sustainability of the work. The burnout rate is really, really high. We know that for every doula we train, only about one in ten is still practicing after a year, let alone multiple years”—Doula D, [California]

## Discussion

By involving doulas in forward-thinking qualitative interviews and workshops, we gained a detailed understanding of how increased technology use in doula care during and after the COVID-19 pandemic could impact the field. This process also led to the identification of potential strategies for ensuring the sustainability of doula practice in the future. In the following section, we will examine the significance of these key findings and put forward recommendations for policy and practice aimed at advancing the future of doula care.

Several theories and frameworks can be applied when discussing the significance of virtual doula communities and their impact on knowledge sharing, peer support, and networking among birth workers. Doula communities housed on social media can be viewed as virtual communities of practice (VCoPs), or online networks of individuals who interact regularly to share their interests and develop their knowledge, skills, and capabilities concerning a particular issue (Wenger-Trayner, 2015). Doulas’ increased reliance on Facebook groups as VCoPs during the COVID-19 pandemic aligns with broader trends showing the global surge in internet and mobile device usage (De’ et al., 2020) and the increased prevalence of VCoPs in healthcare settings (Barnett et al., 2016; Fragou, 2020). The ability of VCoPs to foster connectivity among healthcare workers (Ikioda et al., 2014) is particularly important because it allows doulas, who often work independently or in small groups, to access a broader network of peers, resources, and information regardless of limitations imposed by geography, cost, and organizational boundaries. Prior research suggests that member engagement with VCoPs is strengthened by ensuring active facilitation, a stimulating environment (Ford et al., 2015), and the employment of  technologies that allow for a range of communications and ease of use (McLoughlin et al., 2018). Thus, we suggest technologists center doulas during the development of digital health innovations that may serve as VCoPs, including doula-focused telehealth platforms, virtual doula training programs, doula-specific mobile apps, online forums dedicated to doulas, and remote monitoring and support tools. By centering doulas in the development of these digital health innovations, technologists can ensure that VCoPs are built with a deep understanding of the unique needs, preferences, and expertise of doulas, ultimately leading to more effective and impactful solutions for maternal health.

There may be several advantages to virtual doula care over traditional doula support. Virtual doula support allows doulas to reach a wider range of clients, bringing them into communities that lack them (e.g., rural areas). By doing so, diverse doulas can serve communities that lack workforce diversity, facilitating racially concordant care. Video visits are also less costly and more convenient than in-person visits for both birthing people and the doulas serving them. Virtual support eliminates the need for doulas to spend time and money on travel, allowing them to focus on providing care and support to clients. Virtual doula care also can make doula services more affordable for clients, as there are no additional costs associated with in-person visits.

While virtual doula care may have benefits, like offering accessibility and flexibility for doulas and the communities they serve, there are potential drawbacks to consider. Doulas provide emotional and physical support during labor, and doulas not physically present may pose a challenge, particularly for hands-on comfort measures. Others have also observed that without a physical presence, establishing trust with clients, building rapport with clinicians in the birthing room, and advocating on their client’s behalf can be more challenging (Nguyen et al., 2022). Furthermore, not all birthing people, particularly those from low-income households, rural areas, and individuals with disabilities, have equal access to a reliable internet connection, necessary devices, or technological literacy. Thus, the digital divide presents a significant drawback to virtual doula support and may potentially widen existing healthcare disparities.

Existing literature suggests that empowered birth experiences facilitated by community doula support can lead to improved maternal health outcomes and community well-being in the long term. At the micro level, individuals of color who receive community doula support often report feeling more focused and capable of making decisions during challenging labor experiences, despite encountering complications, and describe positive birth experiences (Arteaga et al., 2023). Promoting positive birth experiences not only benefits individuals and families but also contributes to healthier and more resilient communities in several ways. Compassionate and supportive care during childbirth leads to lower rates of postpartum depression, higher rates of breastfeeding initiation and continuation, and increased satisfaction with the birth process (Kennell et al., 1991; Bohren et al., 2017). These positive outcomes create a ripple effect, strengthening interpersonal relationships within families and communities. When individuals feel empowered and supported during childbirth, they are more likely to extend that support to others, fostering a culture of care and support that transcends individual interactions. Overall, individual positive birth experiences have community-wide positive impacts by fostering resilience, social support, and cultural continuity, ultimately contributing to healthier and more vibrant communities.

To foster intersectional collaboration between healthcare providers and doulas, it is essential to equip healthcare professionals with comprehensive training on the roles of doulas, while also establishing avenues for community building through storytelling and shared experiences. Conflict between doulas and healthcare providers often stems from perceived interference with clinical decision-making, obstruction of medical care, or damage to practitioner-patient relationships (Neel et al., 2019; Papagni & Buckner, 2006). Such conflict may occur due to misunderstandings or misconceptions about the role and scope of doulas within the healthcare system. Therefore, healthcare providers can benefit from comprehensive training on the role of doulas in maternal healthcare. Integrating training modules or workshops on doula care and responsibilities into existing educational curricula for healthcare providers will not only strengthen providers’ understanding of how doulas complement existing healthcare services but also highlight their proven effectiveness in addressing racial disparities in maternal health outcomes. Building connections and fostering empathy between healthcare providers and doulas may also be achieved through digital storytelling. Digital stories are described as short visual narratives that combine and synthesize images, video, audio recordings of voice and music, and text to create engaging and compelling accounts of experience (Gubrium, 2009). Previous work has highlighted a collaborative approach to producing digital stories that can be used to promote discussion and action for clinical quality improvement and healthcare team member training (Behnam Asl et al., 2022). By leveraging existing models, digital storytelling can allow both healthcare providers and doulas to share authentic personal stories, experiences, and perspectives, which can build empathy and understanding between the two groups. Fostering collaboration between healthcare providers and doulas through digital storytelling is one strategy to enhance a birthing person’s perinatal experience, by promoting team building, mutual understanding, and shared best practices among their care team.

As doula care is increasingly being recognized as a vital resource to advance maternal health equity, we suggest that lawmakers and advocates prioritize expanding doula coverage in private insurance plans, as well as public plans, to realize the full benefits associated with doula care. Only one state, Rhode Island, has passed legislation requiring doula coverage in private insurance plans, which is expected to expand access to doula care, improve labor and delivery outcomes, and decrease costs (Chen et al., 2023). Several other states, including California, Indiana, and New York, among others, are also considering expanding private coverage of doula care (Chen et al., 2023). Moreover, doula care is cost-effective, meaning insurance plans can cover doula care without incurring extra expenses (Chapple et al., 2013). In addition to the lack of doula care coverage among public and private insurers, payment models have also been identified as a barrier to implementing community doula programs (Marshall et al., 2022). Lawmakers should introduce legislation mandating private insurance coverage for doula services. To make it easier for doulas to navigate the reimbursement process with insurance companies, policymakers should work with healthcare stakeholders and doulas themselves to establish clear reimbursement guidelines outlining the types of services covered, reimbursement rates, and billing procedures. Lawmakers can also support the development of standardized training and certification programs for doulas. By establishing consistent standards for doula training and certification, policymakers can ensure that doulas are well-prepared to provide high-quality care to clients, which can increase confidence among insurance companies in covering doula services. Thus, private insurance coverage is a crucial step for doula care and is necessary to ensure that all birthing people who want a doula can access one.

Supporting doulas who may experience vicarious trauma is crucial for their well-being and the quality of care they provide. Doulas belong to the category of long-term care workers who often experience elevated rates of burnout (Shin & Moon, 2018; Rachel & Francesco, 2018). Furthermore, results from a survey conducted by Mental Health America (MHC) found that the COVID-19 pandemic exacerbated the challenges faced by healthcare workers, leading to increased stress, exhaustion, and a lack of emotional support among the significant majority (MHC, 2020). With doulas, and healthcare workers at large, facing amplified challenges amid the COVID-19 pandemic, it becomes imperative to propose strategies that bolster emotional support to sustain their crucial work. Previous research suggests female healthcare workers experience significantly higher rates of burnout and depressive symptoms than their male colleagues and low use of mental health services (Sanford et al., 2021). As the doula profession remains female-dominated, ensuring that doulas have access to comprehensive mental health resources is crucial to ensure sustainability in the workforce so birthing persons, especially those coming from vulnerable populations, can continue to benefit from doula support. Establishing regular supervision sessions or peer support groups where doulas can discuss their experiences, share coping strategies, and receive validation from peers is one way to promote community-building among doulas. Additionally, ensuring doulas receive training in trauma-informed care practices, which emphasize safety, trustworthiness, choice, collaboration, and empowerment, can help doulas navigate sensitive situations with greater sensitivity and empathy. We also suggest implementing policies that require doula agencies or organizations to provide mandatory support services, such as accessible and confidential counseling and therapy sessions, for doulas who have experienced vicarious trauma.

*Strengths*: Utilizing a futures-centered approach allowed doulas to create plausible visions for where doula care and childbirth are heading, an outcome that may not have been achieved through conventional interviewing. Futures-thinking served as a valuable tool for doulas to illuminate the implications of present-day challenges: lack of financial support, doula burnout rates, and disconnects between clinicians, and empowered doulas to design roadmaps toward their desirable futures by brainstorming alignment across other stakeholders. Engaging all doulas virtually during a time when travel was limited due to COVID-19 allowed us to speak with a diverse set of doulas from across the United States. *Weaknesses:* Weaknesses of the study included the lack of a representative sample. The number of clients served by participating doulas wasn’t collected, which could have provided a clearer indication of their experience compared to just the number of years they have been practicing. Time limitations during ninety-minute futures workshops limited the depth in which preliminary themes could be further explored during ‘visioning’ and ‘backcasting’ exercises during workshops.

### Recommendations for Future Research

To further explore the increased use of technology in doula care, we suggest speaking with doulas, birthing people, and healthcare professionals to gain insights into the effectiveness, challenges, and opportunities associated with virtual doula communities. Comparative studies can be done to assess the advantages and drawbacks of virtual compared to traditional doula support and examine outcomes such as birth satisfaction, cost-effectiveness, and accessibility while also considering factors like trust-building, rapport with clinicians, and the digital divide. Future research should assess the effectiveness of policies mandating doula coverage in private insurance plans and examine reimbursement processes, coverage disparities, and cost-effectiveness from the insurer and doula perspectives. Future research could focus on the effectiveness of different support mechanisms such as peer support groups, trauma-informed care training, mandatory counseling services, and policy interventions aimed at promoting doulas’ well-being and sustainability in the workforce. Further steps would be to do similar work with a broader group of maternal and child health stakeholders, including but not limited to government agencies, leaders of community-based organizations, healthcare providers, families, and birthing people, as well as folks in the private sector.

## Data Availability

Not applicable.

## Footnotes

1 The term birthing persons includes all people who experience pregnancy and identify as being part of the birthing community.
